# A Quantitative Structure-Activity Relationship and Molecular Modeling Study on a Series of Heteroaryl- and Heterocyclyl-Substituted Imidazo[1,2-a]Pyridine Derivatives Acting as Acid Pump Antagonists

**DOI:** 10.1155/2013/141469

**Published:** 2013-09-08

**Authors:** Neeraj Agarwal, Anubha Bajpai, Satya P. Gupta

**Affiliations:** ^1^Department of Biotechnology, Meerut Institute of Engineering and Technology, Meerut 250005, India; ^2^Department of Pharmaceutical Technology and Department of Applied Sciences, Meerut Institute of Engineering and Technology, Meerut 250005, India

## Abstract

A quantitative structure-activity relationship (QSAR) and molecular docking study has been performed on a series of heteroaryl- and heterocyclyl-substituted imidazo[1,2-a]pyridine derivatives acting as acid pump antagonists in order to have a better understanding of the mechanism of H^+^/K^+^-ATPase inhibition. The QSAR study shows a significant correlation of activity with Global Topological Charge Indices (GTCI) of the compounds and the hydrophobic constant *π* of some substituents, indicating that the charge transfer within the molecule and the hydrophobic property of some substituents will be the controlling factor of the activity of these compounds and that there can be dispersion interaction between the molecules and the receptor, where some substituents may have hydrophobic interaction, too. Based on this correlation some new compounds with higher potency have been predicted and their docking study has been performed to see if they can have better interaction with the receptor. The ADME properties of these predicted compounds have also been reported that follow Lipinski's rule of five.

## 1. Introduction

Gastric H^+^/K^+^-ATPase is a member of the class 2C P-type ion-transport ATPases. It is present in the apical membranes of the parietal cells and is required for acid secretion. Gastric acid is necessary for sterilization and digestion of food and is specially required for the activity of pepsin through the activation of pepsinogen [1]. The H^+^/K^+^-ATPase couples the free energy of ATP hydrolysis for the establishment of the electrochemical gradients for H^+^ across the plasma membrane. Hyperactivity of H^+^/K^+^-ATPase results in overproduction of acid, leading to the gastroesophageal reflux disease (GERD), a condition in which acid leaks into the esophagus from stomach. To treat the hyperacidity and GERD, therefore, the potent inhibitors of H^+^/K^+^-ATPase are desired [2]. H^+^/K^+^-ATPase inhibitors elicit their inhibitory action by binding to the target protein in irreversible manner [3].

The proton pump inhibitors (PPIs) show their inhibitory action against H^+^/K^+^-ATPase by binding to the target protein in irreversible manner [3]. However, there are certain limitations of PPIs in the treatment of GERD and needs some alternative options to cure this disease [2, 3, 5]. Consequently, some potassium-competitive acid blockers (P-CABs), acting as acid pump antagonists, were studied to overcome these limitations of PPIs [2, 6, 7]. P-CABs are more active to achieve faster inhibition of acid secretion and longer duration of action as compared to PPIs, resulting in quicker symptom relief and healing [8]. P-CABs are so called because they block the action of H^+^/K^+^-ATPase by reversible, and K^+^-competitive, ionic binding at the K^+^-binding region of the H^+^,K^+^-ATPase [9]. While PPIs have a unique mechanism of action based on their chemistry, P-CABs have a structural specificity for their target, the K^+^-binding site in the enzyme [10].

From stability point of view P-CABs are significantly more stable at low pH than PPIs. They are lipophilic, weak bases that have high pKa values, because of which they concentrate in acidic medium. On entering an acidic environment, they are instantly protonated to bind with the enzyme. The effect of P-CABs on acid secretion is correlated with plasma concentrations. After oral doses, P-CABs rapidly achieve high plasma concentrations and have linear, dose-dependent pharmacokinetics, and thus inhibit gastric acid secretion with a fast onset of action and have similar effects after single and repeated doses, that is, full effect from the first dose, while PPIs have full effect after repeated doses [10]. Thus, these agents are supposed to produce more rapid acid inhibition and elevate gastric pH to a higher level than PPIs.

**Figure 1:**
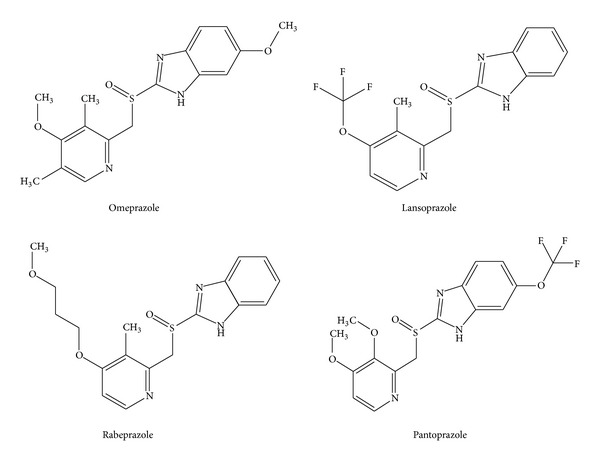
Licensed compounds available in the market to treat the hyperacidity and GERD.

Drug discoveries require the iterative synthesis along with structural studies of numerous individual analogues of biologically and medicinally active compounds. The pharmacological activity of drugs depends mainly on their interaction with their biological targets, which have a complex three-dimensional structure, and the molecular recognition is guided by the nature of intermolecular interactions. QSAR (quantitative structure-activity relationship) approach represents an attempt to correlate the biological activities of compounds with their structural or physicochemical descriptors [11]. Molecular modeling describes the generation, manipulation, or representation of three-dimensional structures of molecules that lead to optimum interactions with the target [11]. We report here a QSAR study on a series of P-CABs to find their physicochemical properties that govern their biological activity and a molecular modeling study to find their three-dimensional mode of interactions with the receptor. An attempt is also made to predict new compounds with better potency based on QSAR model and their ADME properties are reported in accordance with Lipinski's rules that help us to select the biologically active molecules with least adverse effects. Currently, there are only some PPIs that are licensed to treat the hyperacidity and GERD and they are omeprazole, lansoprazole, rabeprazole, and pantoprazole (Figure 1), out of which, omeprazole was the first PPI to reach the market in 1988 and whose properties are well documented [12].

QSAR studies on some series of H^+^/K^+^-ATPase inhibitors and essential information generated can be employed for designing new potent inhibitors and the interactions of these newly designed inhibitors are found with the help of docking studies. Some important QSAR studies on PPIs have been reported in the past. Ojha and coworkers [13, 14] reported excellent QSAR studies on two different series of analogues of omeprazole itself (**1**, **2,** Figure 6). These authors also reported QSARs on a series of 2,3-dihydropyrroloquinolines (**3,** Figure 6) [14] and two different series of 2-guanidinothiazoles (**4**, **5,** Figure 6) [15]. In all their QSAR studies, these authors found the significant role of electronic properties of substituents, indicating that the overall electronic properties of molecules may be important for the inhibition of H^+^/K^+^-ATPase. On the same two series of 2-guanidinothiazoles (**4**, **5**, Figure 6), Grünheidt and Takahata also reported in their two consecutive studies [16, 17] that the electronic properties of compounds such as dipole moment and charges at some atoms are important for their activity. On a fairly large series of *α*-amino acid derivatives, a QSAR study performed by Sharma et al. [18] also suggested that their PPI inhibition activity is controlled by the electronic properties, such as the energy of the highest occupied molecular orbital (*E*_HOMO_), H-bond formation ability of some groups, and steric factors. A series of 179 quinoline and quinazoline heterocyclic analogues exhibiting inhibitory activity against H^+^/K^+^-ATPase was investigated by comparative molecular field analysis (CoMFA) and comparative molecular similarity indices analysis (CoMSIA) by Nayana et al. [3] to find that in addition to electrostatic and steric fields, hydrophobic and H-bond donor and acceptor fields were also important for the H^+^/K^+^-ATPase inhibition activity of these compounds. Recently, we also found for a series of biarylimidazole derivatives that H^+^/K^+^-ATPase inhibition predominantly involves only electronic interaction [19].

## 2. Materials and Methods

A series of thirty-four heteroaryl- and heterocyclyl-substituted imidazopyridine derivatives reported to have gastric H^+^/K^+^-ATPase inhibitory activity were synthesized and evaluated for their antisecretory activity by Bailey et al. [4]. This series is listed in Table 1 along with antisecretory activity of the compounds. Table 1 also lists the physicochemical and topological parameters of the compounds that were found to govern their potency. The values of the hydrophobic constant (*π*) of substituents were taken from the literature [11] and the topological parameter, global topological charge indices (GTCI), has been calculated with the help of E-Dragon, version 1.0 [20].

For performing docking studies and to check the interactions between the predicted compounds and the protein taken from protein data bank (PDB id 2XZB), Molegro Virtual Docker software (trial version) has been employed. To check if the predicted molecules will have acceptable absorption, distribution, metabolism, and excretion (ADME) properties, Lipinski's parameters, that is, molecular weight (MW), number of hydrogen-bond donors (HD), number of hydrogen-bond acceptors (HA), and octanol-water partition coefficient (log *P*), were calculated with the help of Marvin Sketch.

## 3. Results and Discussion

### 3.1. QSAR Results

All the compounds of Table 1 were divided into two subsets: training set and test set. Compounds for the test set were selected arbitrarily by keeping in mind the wide variation in their structures and a good span in their activity data. All the test set compounds are given with superscript “b” and in bold in Table 1. The rest of the compounds were taken in the training set. When a multiple regression analysis was performed on the compounds of the training set, it revealed the following correlation:

(1) log⁡(1IC50)=2.204(±0.888)πo−13.954(±9.026)GTCI+1.313(±0.391)I1+1.328(±0.548)I2+9.964(±3.595),n=24,  r=0.907  rcv2=0.685,rpred2=0.524,  s=0.41,  F4,19=21.90(4.50),

where *π*_*o*_ refers to the hydrophobic constant of left-side ortho-substituent (RHS) at pendent phenyl ring, GTCI is global topological charge indices of the molecule which describes the charge transfer between pairs of atoms, and *I*_1_ and *I*_2_ are two indicator variables that have been used for the variation of methyl-substituents in the compounds. The *I*_1_ has been used with a value of 1 for the presence of a methyl group at right-side ortho-position of the pendent phenyl ring and *I*_2_ has been used with a value of 1 for both the substituents present at imidazo[1,2-a]pyridine ring being a methyl group. In the biological activity term, IC_50_ refers to molar concentration of the compound leading to 50% inhibition of the enzyme.

Among the statistical parameters in (1), *n* is the number of data points, *r* is the correlation coefficient, *r*_cv_^2^ is the square of the cross-validated correlation coefficient obtained from leave-one-out (LOO) jackknife procedure, *s* is the standard deviation, *F* is the Fischer ratio between the variances of calculated and observed activities, and the data within the parentheses with ± sign are 95% confidence intervals. The figure within the parenthesis for *F* is the standard *F*-value at 99% level. The values of these statistical parameters exhibit that the correlation obtained is quite significant. This correlation suggests that the H^+^/K^+^-ATPase inhibition activity of this series of compounds is basically controlled by the hydrophobic property of substituent at the left-side ortho-position of the phenyl ring and global topological charge indices (GTCI) of the molecules. However, while the coefficient of hydrophobic constant *π*_*o*_ is positive, that of GTCI is negative, suggesting that whereas the increase in hydrophobic value of *o*-substituent will increase the activity, the increase in GTCI value of the compound will decrease it. Since GTCI describes the charge transfer between pairs of atoms, the negative coefficient would indicate that excessive charge transfer will not be conducive to the activity and this therefore leads to assume that there may be some electronic interaction between the drug molecule and the receptor, where excessive charge distribution may create some repulsive interaction. The positive coefficients of both the indicator parameters *I*_1_ and *I*_2_ indicated the importance of the presence of methyl groups at both the pendent phenyl ring and imidazo[1,2-a]pyridine ring. These methyl groups may have hydrophobic or steric interactions with some small sites of the receptor.

**Figure 2:**
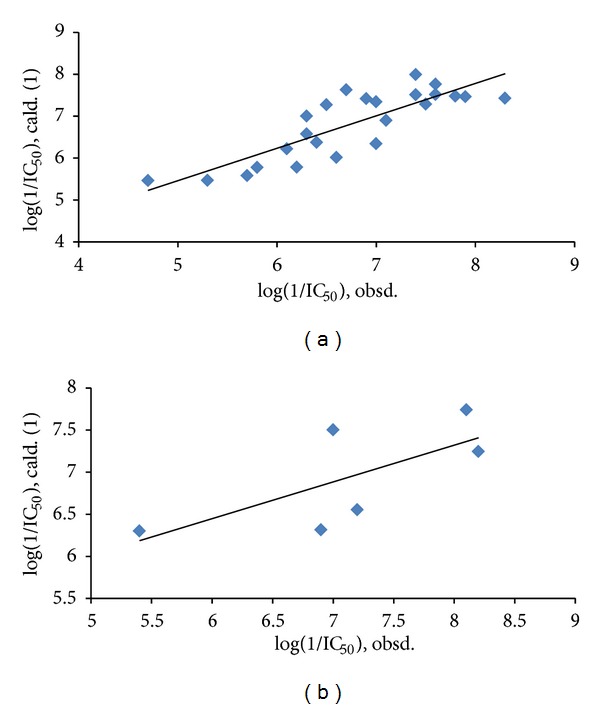
A plot between observed and predicted H^+^/K^+^-ATPase inhibition activities of compounds of Table 1: (a) for training set; (b) for test set.

The correlation expressed by (1) seems to be highly significant and its internal and external validation can be judged by *r*_cv_^2^ and *r*_pred_^2^ values. The *r*_cv_^2^ is calculated according to the formula

(2) $$\begin{array}{c} r_{\text{cv}}^{2}=1-\;\left[\frac{\sum_{i}^{}{\left(y_{i,\text{obsd}}-y_{i,\text{pred}}\right)}^{2}}{\sum_{i}^{}{\left(y_{i,\text{obsd}}-y_{\text{av},\text{obsd}}\right)}^{2}}\right], \end{array}$$

where *y*_*i*,obsd_ and *y*_*i*,pred_ are the observed and predicted (from LOO) activity values of compound *i*, respectively, and *y*_av,obsd_ is the average of the observed activities of all compounds used in the correlation. The correlation is supposed to be valid if *r*_cv_^2^ > 0.60. From this point of view, the correlation expressed by (1) seems to be quite valid. However, the predictive ability of any model is judged by predicting the biological activity of the compounds in the test set using it and calculating the value of *r*_pred_^2^, which is defined as follows:

(3) $$\begin{array}{c} r_{\text{pred}}^{2}=1-\left[\frac{\sum_{i}^{}{\left(y_{i,\text{obsd}}-y_{i,\text{pred}}\right)}^{2}}{\sum_{i}^{}{\left(y_{i,\text{obsd}}-y_{\text{av},\text{obsd}}\right)}^{2}}\right], \end{array}$$

where *y*_*i*,obsd_ and *y*_*i*,pred_ refer to the observed and predicted biological activity values of compound in the test set and *y*_av,obsd_ is same as in (2). The biological activity values predicted from this equation for the test set compounds are given (in bold) in Table 1. A comparison shows that these predicted values are in very good agreement with the corresponding observed ones. In the training set also, the calculated values are found to be in excellent agreement with the observed ones. All these observations can be better visualized in the graphs drawn between the predicted and observed biological activities for both the sets (Figure 2). Using (1), we have predicted the biological activity of some new prospective compound with high potency (Table 2). The activities of these compounds are higher than any compound in the present series (Table 1).

### 3.2. Docking Results

**Figure 3:**
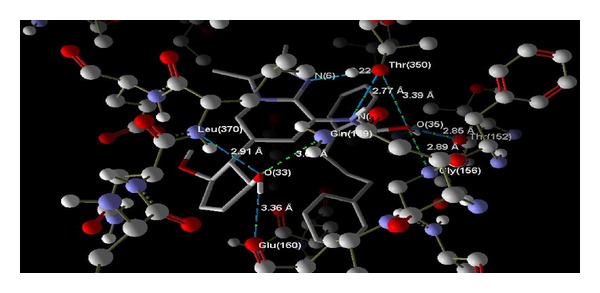
A model showing hydrogen-bond interactions of predicted compound 2 (Table 2) with the enzyme H^+^/K^+^-ATPase. Compound 2 is one of the compounds that have the highest number of H-bonds.

**Figure 4:**
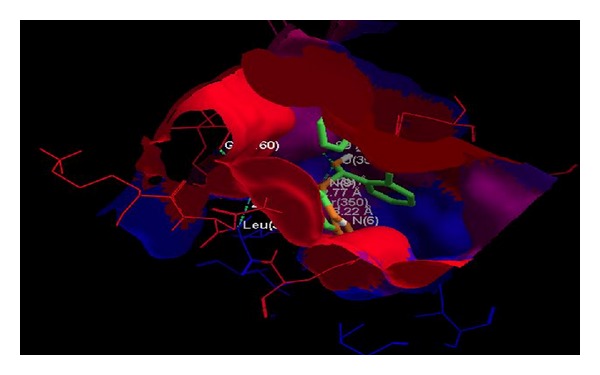
The model showing hydrophobic interactions of predicted compound 2 (Table 2) with the enzyme H^+^/K^+^-ATPase. The red surface shows strong hydrophobic zone and blue one the low hydrophobic zone.

**Figure 5:**
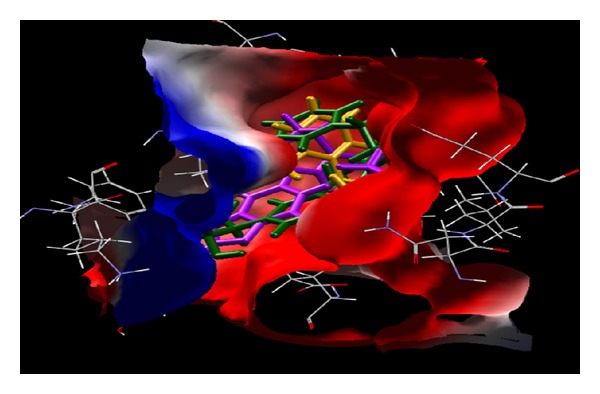
A model showing the binding modes of a compound of training set (compd 3, Table 1), a compound of test set (compd 4, Table 1), and a compound of predicted set (compd 2, Table 2) in purple, yellow, and green, respectively. The red surface shows strong hydrophobic zone and blue one the low hydrophobic zone.

**Figure 6:**
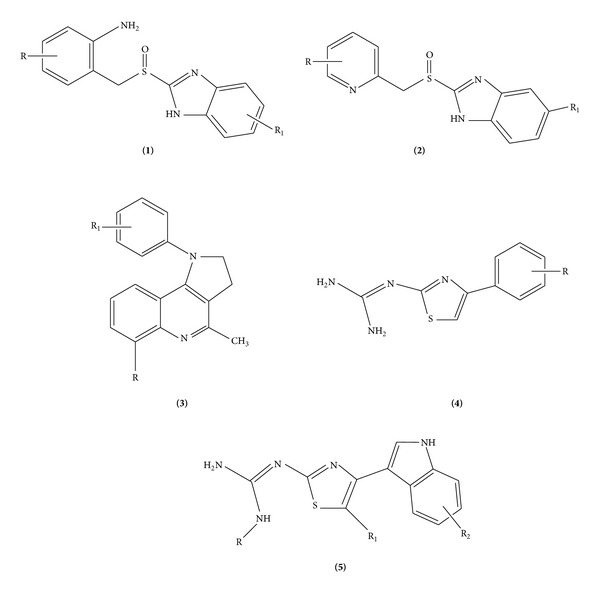


Docking studies on all predicted compounds were performed using Molegro Virtual Docker (trial version) and the results are given in Table 3. As can be seen, the overall energy of interaction with the enzyme for each predicted compound is better than that for any marketed compound and so is the case with the docking score of each predicted compound.  Table 4 shows that all the predicted compounds also obey Lipinski's rules and are thus less likely to produce any ADME problems. According to this rule, an orally active drug, in general, should have no more than one violation of the following criteria: it should not have (1) more than 5 hydrogen bond donors (nitrogen or oxygen atoms with one or more hydrogen atoms), (2) more than 10 hydrogen bond acceptors (nitrogen or oxygen atoms), (3) a molecular mass more than 500 Da, and (4) an octanol-water partition coefficient (log *P*) greater than 5.  Figure 3 shows the hydrogen bond interaction of one of the predicted compounds (compound 2) that have the highest number of hydrogen bondings with the enzyme. For all the compounds, all possible hydrogen bonds are shown in Table 3.  Figure 4 shows the possible hydrophobic interactions of a representative compound (compound 2).  Figure 5 shows a comparative mode of interactions of this compound with a training set compound (compd 3, Table 1) and a test set compound (compd 4, Table 1). All compounds have a matching pose.  Table 5 shows that all the docking results of the predicted compound are much superior to those of training and test set compounds in question.

## 4. Conclusion

This study thus shows that the heteroaryl- and heterocyclyl-substituted imidazo[1,2-a]pyridine derivatives acting as H^+^/K^+^-ATPase inhibitors may inhibit the enzyme through some electronic interaction with the enzyme and some of their small substituents may participate in hydrophobic interaction as well as steric interactions. Based on the correlations obtained, some new compounds in the series have been predicted whose activity is higher than the compounds marketed. The predicted compounds have better docking scores than training or test set compounds. These compounds can be synthesized and tested.

## Figures and Tables

**Table 1:** Heteroaryl- and heterocyclyl-substituted imidazo[1,2-a]pyridine derivatives and their physicochemical parameters and H^+^/K^+^-ATPase inhibition activity.

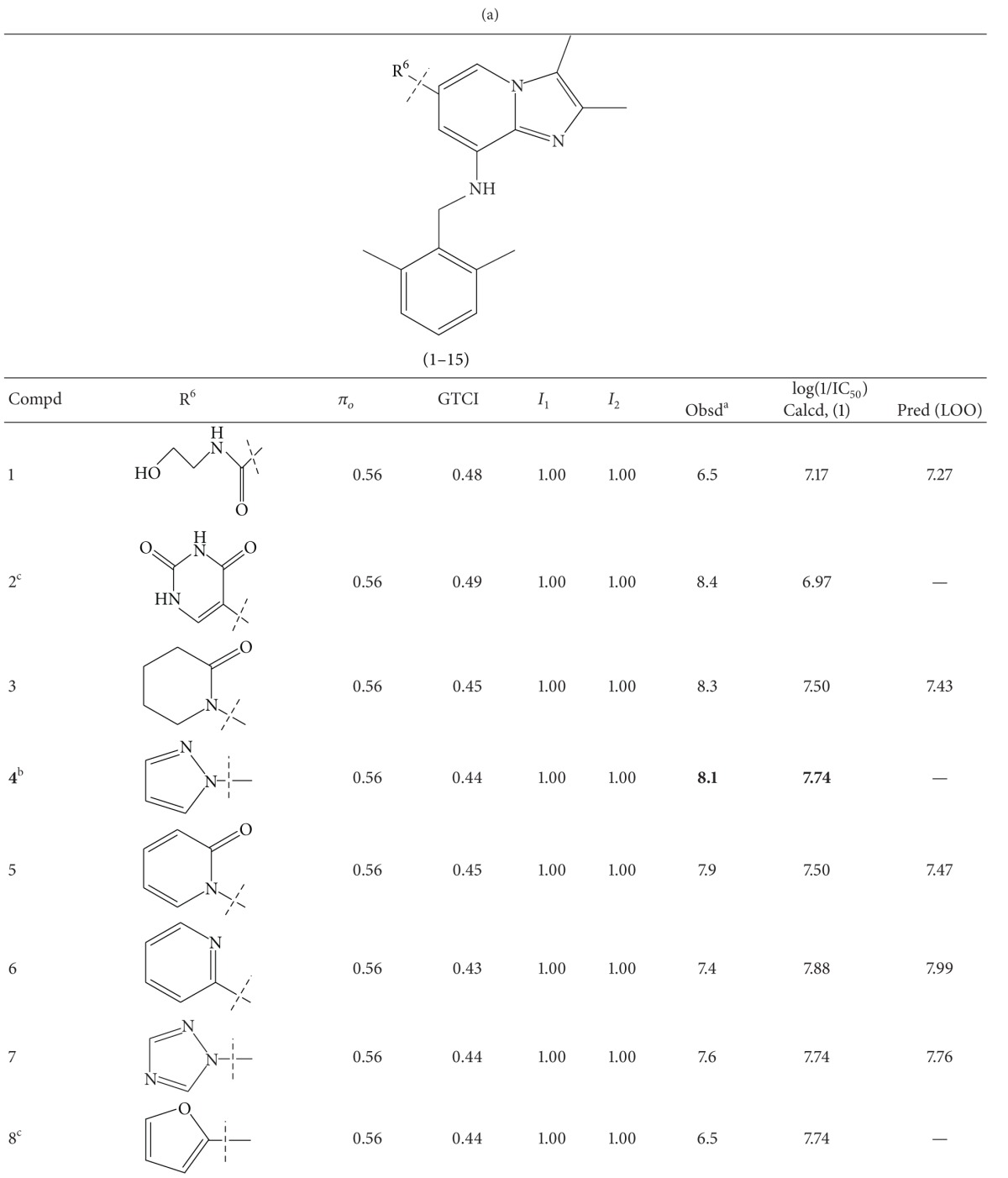 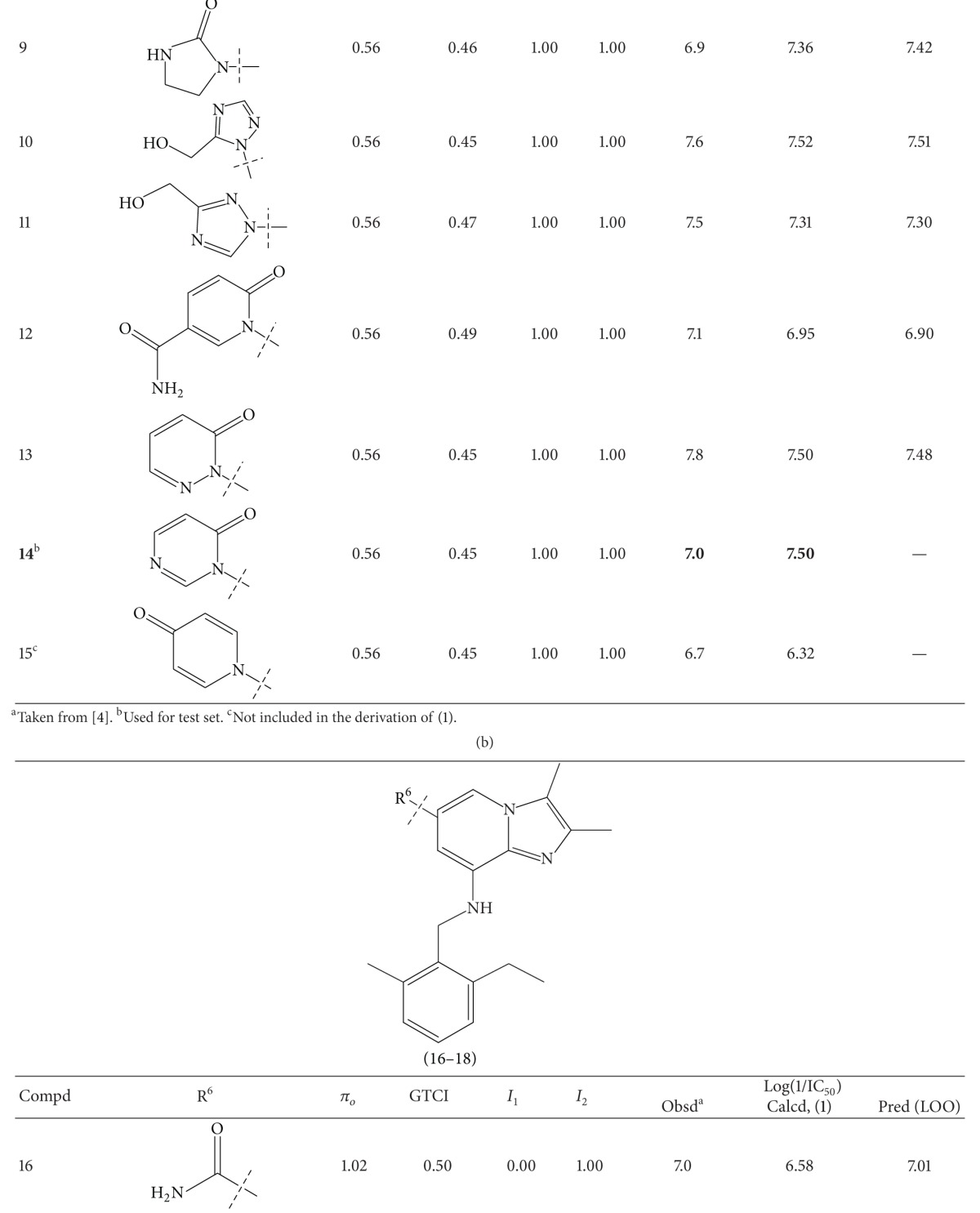 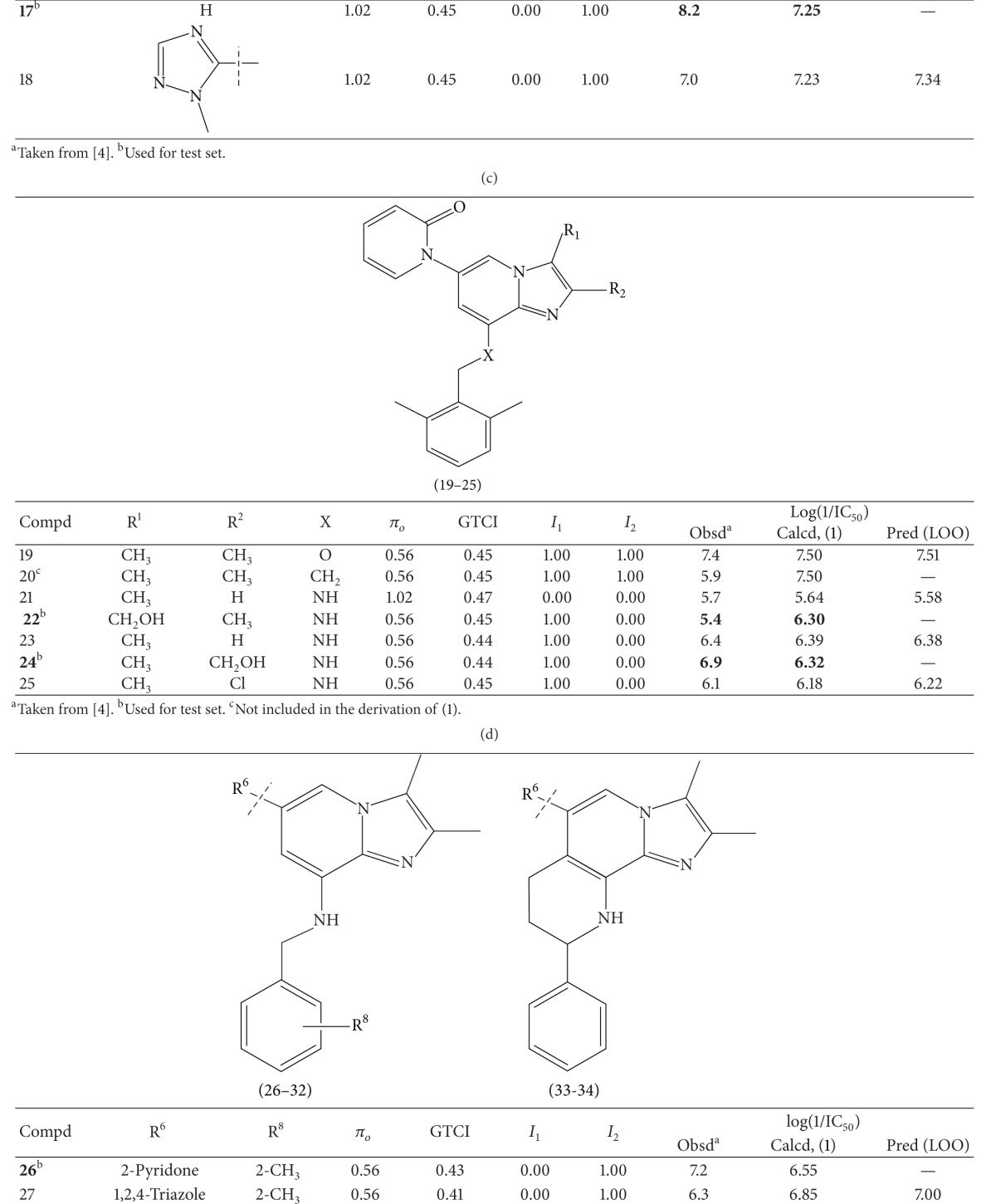 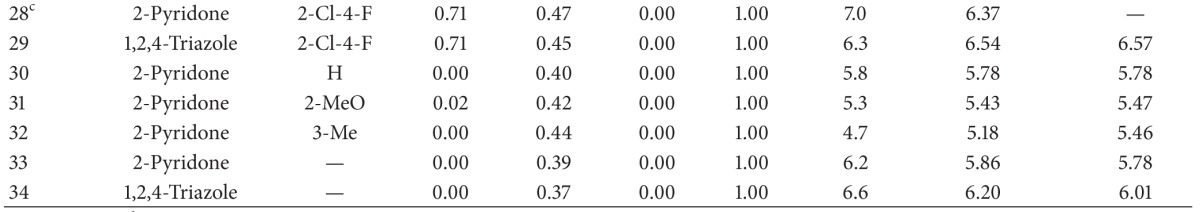

^a^Taken from [4]. ^b^Used for test set. ^c^Not included in the derivation of (1).

**Table 2:** Some proposed compounds belonging to the series of Table 1 and their activities predicted from (1).

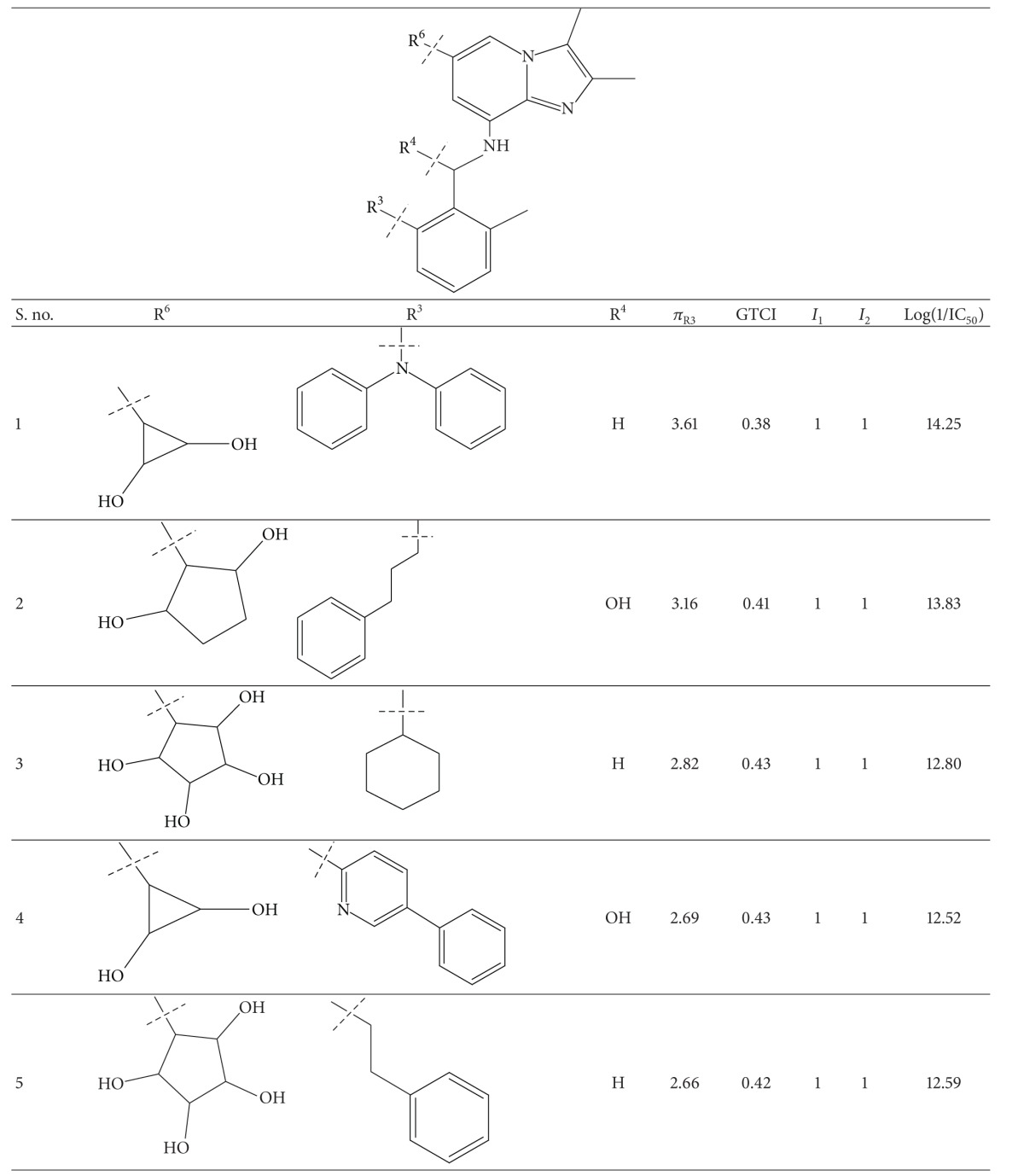 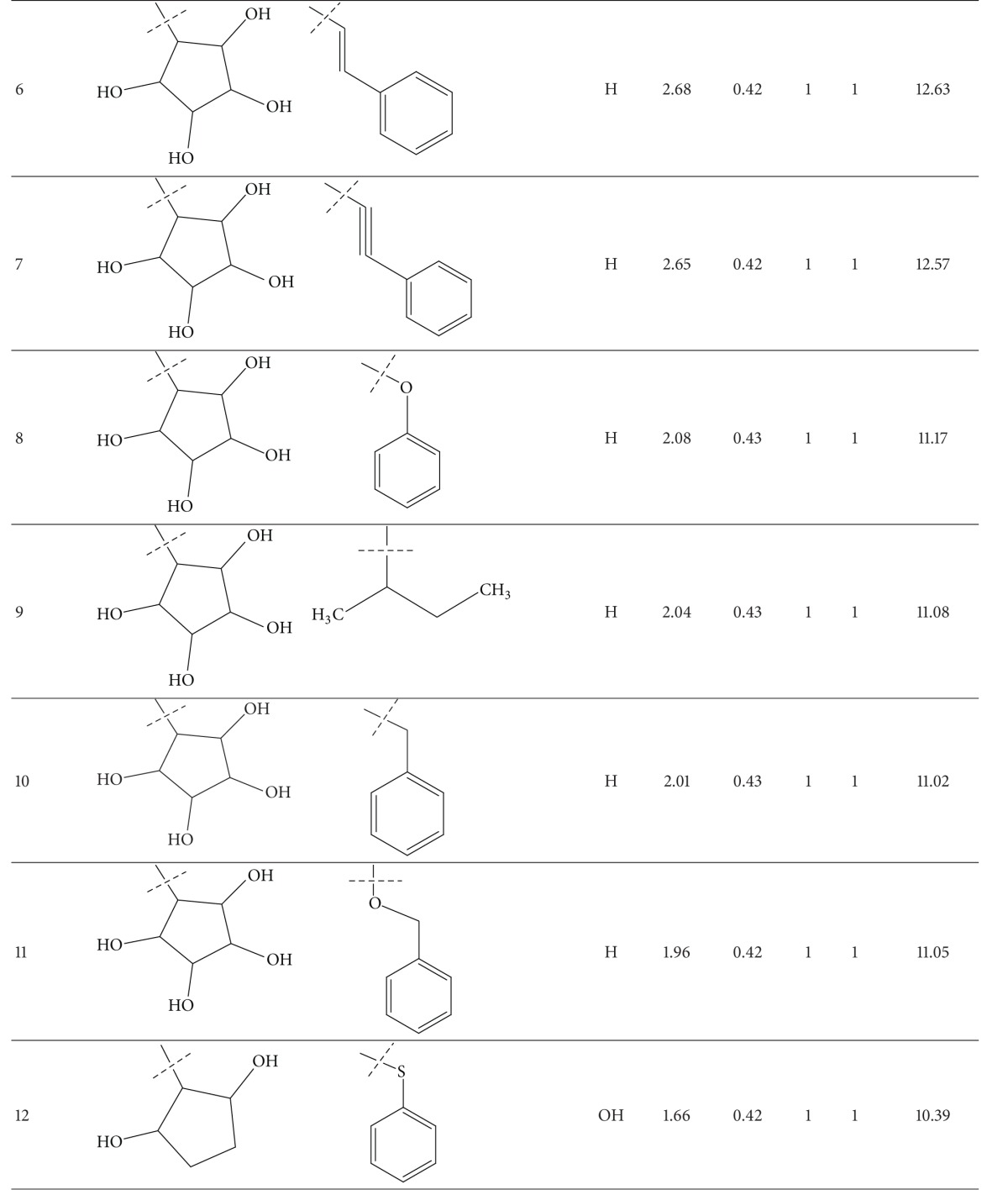 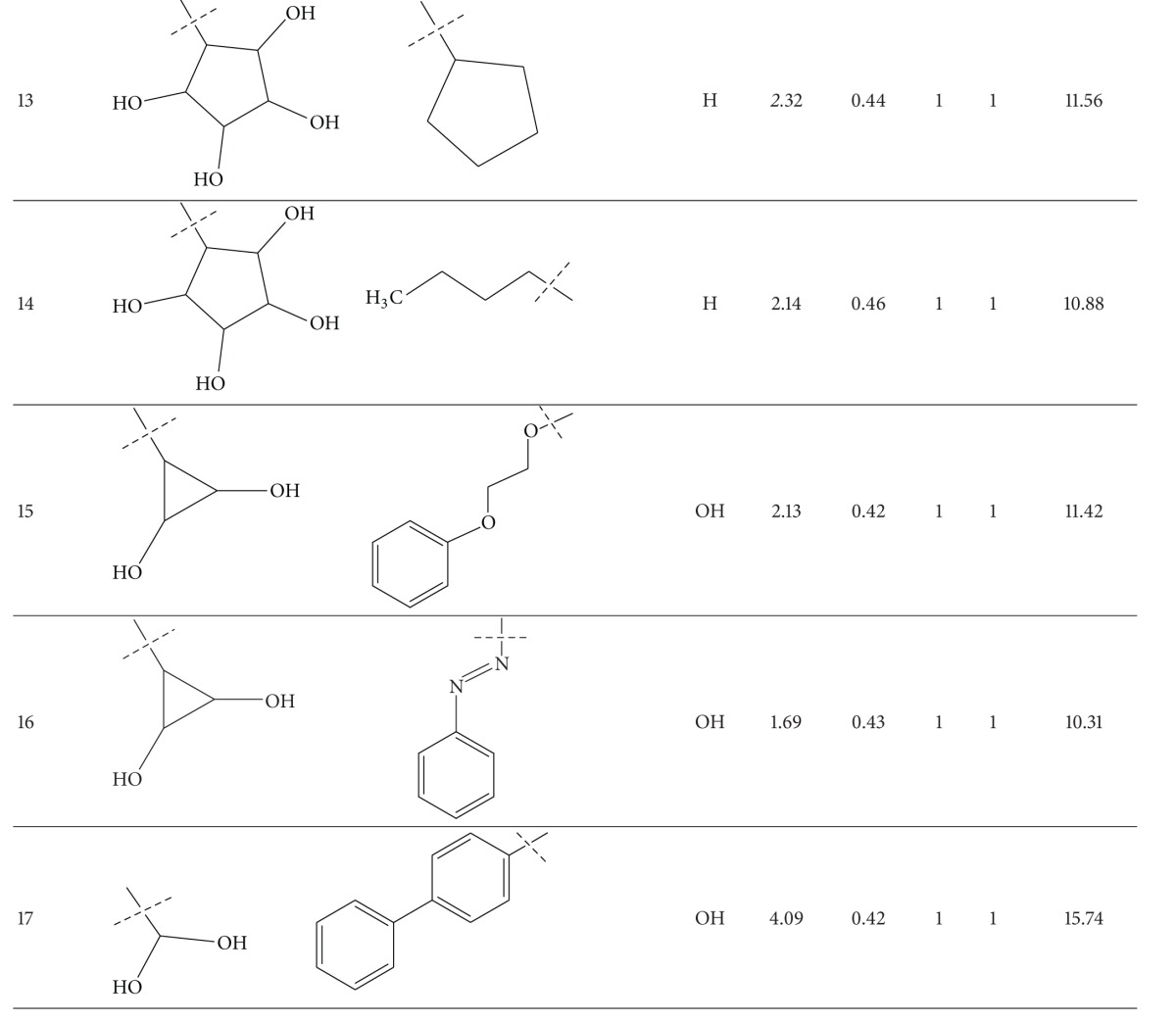

**Table 3:** Docking results of predicted compounds with reference to the active drugs available in the market (last four in the table) to treat the hyperacidity and GERD. Energy values are in kJ/mol.

Pred compd (Table 2)	Total inter. energy	H-bond energy	No. of H-bonds	H-bonds ligand-protein	H-bond length (Å)	Mole Dock score	Inter. E of pose
1	−175.83	−0.91	1	O(35)-Glu(97)	3.42	−176.36	−0.53

2	−178.33	−10.33	8	O(35)-Thr(350) O(35)-Gly(156) O(35)-Thr(152) N(9)-Thr(350) N(6)-Thr(350) O(33)-Gln(159) O(33)-Leu(370) O(33)-Glu(160)	3.39 2.89 2.85 2.77 3.32 3.60 2.91 3.36	−197.72	−19.39

3	−179.52	−10.06	5	N(6)-Thr(350) N(9)-Thr(350) O(34)-Thr(350) O(34)-Thr(152) O(32)-Leu(370)	3.26 2.67 3.42 2.17 3.08	−180.64	−1.12

4	−148.24	−14.38	6	O(36)-Asn(369) O(35)-Gln(159) O(34)-Thr(350) O(34)-Thr(352) N(9)-Thr(350) N(6)-Thr(350)	2.68 3.35 3.38 2.66 2.72 3.23	−168.07	−19.83

5	−191.53	−11.70	3	O(32)-Gly(107) O(33)-Gly(107) O(35)-Leu(346)	2.86 2.93 2.49	−191.78	−0.25

6	−154.31	−6.58	2	O(32)-Leu(346) O(32)-Thr(350)	2.51 3.26	−177.77	−23.46

7	−176.30	−2.04	3	O(34)-Thr(350) O(33)-Thr(350) O(33)-Leu(346)	3.23 3.26 2.95	−190.78	−14.48

8	−172.98	−4.38	6	O(34)-Thr(350) O(32)-Gln(159) O(33)-Gly(156) O(33)-Glu(160) O(32)-Leu(370) N(6)-Gln(110)	2.75 2.72 2.99 3.22 2.95 3.56	−183.57	−10.59

9	−152.69	−9.56	4	O(28)-Asn(369) O(30)-Glu(371) O(30)-Leu(370) O(31)-Glu(160)	3.19 3.15 2.60 3.12	−170.18	−17.48

10	−146.03	−8.27	8	N(6)-Tyr(157) N(9)-Gly(153) O(31)-Gln(104) O(34)-Glu(160) O(34)-Leu(370) O(33)-Glu(160) O(33)-Asn(369) O(32)-Asn(369)	3.53 2.71 2.77 2.94 3.33 3.05 2.61 3.03	−150.52	−4.50

11	−165.10	−15.21	0	—	—	−179.73	−14.63

12	−147.86	0	6	O(33)-Gln(104) O(33)-Lys(100) O(32)-Gly(153) O(27)-Glu(153) O(27)-Thr(152) O(27)-Gly(156)	3.13 3.14 2.67 2.90 3.27 3.00	−173.20	−25.33

13	−165.88	−11.84	6	N(9)-Thr(150) N(6)-Thr(150) O(32)-Gln(104) O(29)-Gln(104) O(30)-Asn(369) O(31)-Gln(160)	2.49 3.10 2.92 3.09 2.84 2.79	−167.27	−1.38

14	−149.50	−13.62	8	N(9)-Gly(153) O(30)-Gln(104) O(30)-Gln(104) O(31)-Leu(370) O(28)-Glu(160) O(31)-Glu(160) O(28)-Lys(368) O(29)-Asn(369)	2.60 2.65 3.21 3.11 2.68 3.32 3.26 2.66	−170.60	−21.10

15	−161.87	−14.80	6	O(34)-Gln(104) O(33)-Glu(160) O(32)-Gly(156) O(32)-Thr(152) N(6)-Thr(350) N(9)-Thr(350)	2.96 3.12 2.90 2.66 3.43 2.73	−168.46	−6.58

16	−177.11	−11.22	4	O(31)-Gln(104) O(30)-Thr(152) O(32)-Leu(370) O(32)-Glu(160)	2.87 3.08 3.19 3.14	−179.76	−2.65

17	−150.98	−9.31	5	O(32)-Leu(370) O(34)-Thr(350) N(6)-Thr (350) N(9)-Thr(350) O(35)-Thr(152)	3.08 3.42 3.26 2.67 2.71	−159.32	−8.34

Rabeprazole	− 144.85	−5.00	2	O(11)-Thr(350) N(13)-Thr(350)	2.63 3.01	−131.76	13.09

Lansoprazole	−121.59	−0.10	2	O(19)-Gly(156) N(8)-Gln(104)	3.48 3.59	−115.07	6.521

Omeprazole	−117.83	−2.42	2	O(24)-Arg(103) O(24)-Arg(103)	2.75 3.34	−111.99	5.84

Pantoprazole	−120.43	−3.78	3	O(21)-Thr(350) N(6)-Gln(104) O(11)-Gln(110)	3.22 3.10 2.99	−115.41	5.02

**Table 4:** Data related to Lipinski rules in comparison to those licensed.

Predicted compound (Table 2)	Lipinski parameters			
	MW	HA	HD	log *P *
1	504.622	6	3	4.65
2	499.644	6	4	4.43
3	479.611	7	5	2.09
4	506.594	7	4	3.34
5	501.616	7	5	2.52
6	499.600	7	5	2.32
7	497.584	7	5	2.10
8	489.562	7	5	1.48
9	453.573	7	5	1.67
10	487.590	7	5	2.07
11	503.589	8	5	1.55
12	489.629	6	4	3.64
13	465.584	7	5	1.65
14	453.573	7	5	1.83
15	489.562	8	4	2.36
16	457.564	8	4	3.28
17	479.569	6	4	4.60
Rabeprazole	339.388	5	1	2.56
Lansoprazole	369.361	7	1	3.03
Omeprazole	383.370	8	1	2.18
Pantoprazole	345.416	5	1	2.43

**Table 5:** Docking results of a training set compound (3), a test set compound (4) of Table 1, and a predicted compound (2) for comparison. Energy values are in kJ/mol.

Compd	Total inter. energy	H-bond energy	No. of H-bonds	H-bonds ligand-protein	H-bond length (Å)	Mole Dock score	Inter. energy of pose
3 (Table 1)	−139.62	−0.627	1	O(27)-Gly(156)	2.79	−139.78	−0.19

4 (Table 1)	−131.97	−4.752	1	N(26)-Thr(350)	2.96	−139.73	−7.76

2 (Table 2)	−178.33	−10.33	8	O(35)-Thr(350) O(35)-Gly(156) O(35)-Thr(152) N(9)-Thr(350) N(6)-Thr(350) O(33)-Gln(159) O(33)-Leu(370) O(33)-Glu(160)	3.39 2.89 2.85 2.77 3.32 3.60 2.91 3.36	−197.72	−19.39
